# Task Monitoring and Working Memory as Executive Components Predictive of General and Specific Academic Achievements in 6–9-Year-Old Children

**DOI:** 10.3390/ijerph18136681

**Published:** 2021-06-22

**Authors:** Alberto Quílez-Robres, Nieves Moyano, Alejandra Cortés-Pascual

**Affiliations:** 1 Department of Educational Sciences, Faculty of Humanities and Educational Sciences, University of Zaragoza, 50001 Zaragoza, Spain; 2 Department of Psychology and Evolutionary Education, Faculty of Humanities and Educational Sciences, University of Jaén, 23009 Jaén, Spain; mnmoyano@ujaen.es; 3 Department of Education Sciences, Faculty of Education, University of Zaragoza, 50001 Zaragoza, Spain; alcortes@unizar.es

**Keywords:** executive functions, working memory, task monitoring, academic achievement, learning

## Abstract

Academic achievement has been linked to executive functions. However, it is necessary to clarify the different predictive role that executive functions have on general and specific academic achievement and to determine the most predictive executive factor of this academic achievement. The relationship and predictive role between executive functions and their components (initiative, working memory, task monitoring, organization of materials, flexibility, emotional control, inhibition, self-monitoring) with academic achievement are analyzed in this study, both globally and specifically in the areas of Language Arts and Mathematics, in 133 students from 6 to 9 years of age. The relationship obtained in Pearson’s correlation analysis does not differ substantially between overall achievement (r = 0.392) and specific achievement (r = 0.361, r = 0.361), but task monitoring (r = 0.531, r = 0.455, r = 0.446) and working memory (r = 0.512, r = 0.475, r = 0.505) had a greater relationship with general and specific achievement. Finally, regression analyses based on correlation results indicate that executive functions predict general academic performance (14.7%) and specific performance (12.3%, 12.2%) for Language Arts and Mathematics, respectively. Furthermore, working memory and task supervision represent 32.5% of general academic performance, 25.5% of performance in Language Arts, and 27.1% of performance in Mathematics. In conclusion, this study yielded exploratory data on the possible executive functions (task supervision and working memory) responsible for good general academic achievements and specific academic achievements in Mathematics and Language Arts.

## 1. Introduction

The educational community has shown special interest in the study of academic achievement over time. In this field, academic achievement is defined as the product of the learning process resulting from the different variables or factors that influence that process [1,2]. As academic achievement can be understood as quantitative or qualitative value construction related to a profile of skills, attitudes, and knowledge developed by the student in the teaching and learning process [3], the relevant skills and cognitive factors are of great importance for understanding the correct progress of this process [4]. Within the discipline of educational neuropsychology, there are those who highlight the need to study the influence that executive functions have on academic achievement as a consequence of their importance on language development and, therefore, on literacy as a gateway to knowledge [5].

**Figure 1:**
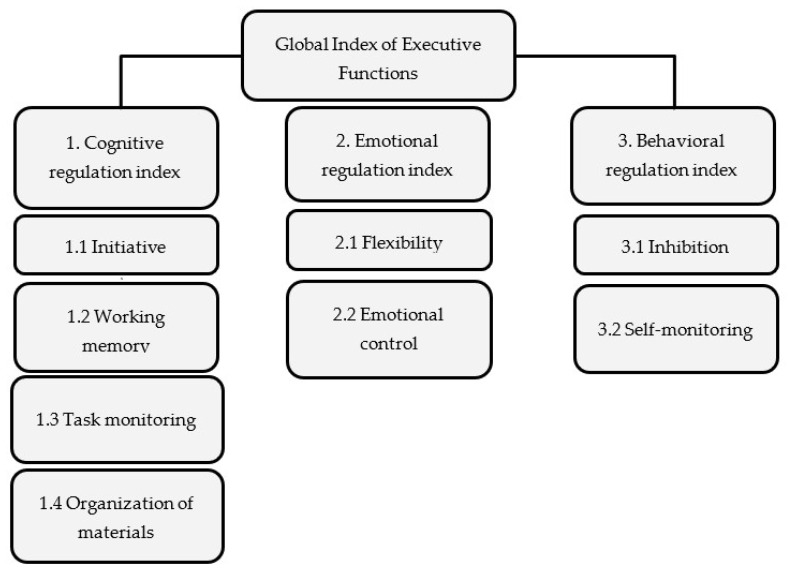
Scheme of indexes and scales of the executive functions according to Gioia et al. Source: own preparation.

The executive functions are understood as a multifactorial structure that intervenes in different important neurocognitive processes and controls thoughts and behaviors to achieve an objective [6,7,8]. Therefore, these functions act as regulators of cognitive activity and behavior through their special relationships with intrapersonal intelligence [9], as executive processes do not refer only to cognitive processes (cold executive functions) but also to emotional and behavioral ones (warm executive functions) [10]. Several authors have highlighted inhibition, working memory, flexibility, and planning as factors or components of executive functions [11,12,13]. However, Gioia et al. [14] proposed three indices (of behavioral, emotional, and cognitive regulation) grouped together with nine scales: inhibition or impulse control; self-monitoring or awareness of one’s own behavior; cognitive flexibility or the capacity to generate different solutions to the same problem; emotional control, understood as the capacity to regulate one’s emotional response; the initiative to act autonomously and independently; working memory, understood as the capacity to manipulate information temporarily; planning, or the development of strategies to achieve objectives; task supervision, such as the capacity to review and evaluate; and, finally, the organization of materials or the capacity to order (Figure 1).

Executive functions have a significant impact on academic results from preschool to university, with the executive level recorded at a specific age being related to results obtained at later ages and replacing the importance of one executive factor with another [15,16,17]. A review of the components of the executive functions can provide information on the involvement of such functions in scholastic achievement [14]. The behavioral regulation index includes inhibition and self-monitoring. Inhibition or the control of one’s behavior is understood as the ability to suppress dominant and impulsive responses that are irrelevant to the task at hand [18,19,20,21,22]. Self-supervision, in a broad sense, refers to the ability to plan voluntarily, modulating one’s behavior adaptively as needed to meet the established plan. The ability to self-regulate one’s behavior is acquired between the ages of 6 and 8 and allows for the anticipation of events, even if a certain degree of impulsivity persists [19,20]. Because of its link to behavior and cognition, inhibition plays a significant role in the achievements of younger children [11,21,22,23]. It is also considered to be a good predictor of academic achievement up to age seven, since from three to seven years of age, a change occurs as the child progresses toward cognitive behavioral forms that require the integration of executive functions with linguistic skills [24,25,26,27,28]. However, this claim lacks strong consistency and has as many supporters as detractors. This variation of skills in early childhood predicts a multitude of results, among which the academic achievements in the Primary Education stage stand out [29]. These differences are explained according to the moment at which this integration takes place since such differences will depend on the child’s development [30]. There is also a close relationship between inhibition and self-control or self-supervision, to academic achievement [31], and its variation with age [32,33].

Both flexibility and emotional control comprise the index of emotional regulation [14]. Flexibility involves the choice of appropriate work strategies and the ability to change the focus of one’s attention [34,35,36]; this factor is, therefore, related to good academic achievement [37] in both Mathematics [38] and Language Arts [39], but in a less concrete way in the latter discipline [40]. There is evidence that academic achievement improves at the age of 5 or 6 and becomes significant by the age of 7–11 [41,42]. However, “emotional control evaluates the presence of problems in order to adequately regulate or modulate their emotional responses” [14] (p. 12). Emotional control is related to flexibility in assessing emotional responses during changing situations. Emotional control affects individual academic achievement as it can be a stress-inducing factor [43]. However, some studies assign control only a limited role in academic success [44].

The cognitive regulation index is composed of initiative, working memory, planning, task supervision, and the organization of materials [14]. Initiative “evaluates the presence of problems in order to initiate tasks or activities in an autonomous and independent way or to generate new ideas, answers or problem-solving strategies” [14] (p. 51). In other words, initiative is the ability to act without the need for external motivation to direct one’s behavior. Deficits in this component may indicate difficulties in verbal and visual fluency, which will lead to low academic achievement [45].

Working memory represents the capacity to store, retain, and retrieve previous information and can be defined as a multifactorial memory system involved in the coordination and regulation of executive control and selective attention functions [46,47]. Various studies have concluded that working memory is the most relevant component of the executive functions, increasing its importance in the achievement of complex tasks. Working memory is also a good predictor of academic achievement during the first years of compulsory schooling [35,41,48] and presents early development that improves with age until 10 years old [49,50,51]. This factor has a good relationship with academic achievement in specific subjects such as Language Arts and Mathematics [17,52,53].

Baggetta and Alexander [54] identified planning as a higher order cognitive process necessary for the anticipation and execution of a task in the correct manner using the appropriate strategies. Within the factorial order of executive functions, planning coordinates various processes of analysis, selection, and the application of information and strategies necessary to achieve an objective. In this way, achieving good academic results indicates adequate executive functioning for the correct identification of the problem and its definition, the search for solutions, and the planning of an execution plan [11,49]. There is consensus that the components of inhibition (the scale of the behavioral regulatory index) and planning complement each other and are necessary both to solve a written text and to perform a mathematical calculation. These components are also strong predictors of specific achievements such as Language Arts or Mathematics [38,40,55].

Task supervision and self-monitoring have traditionally been considered using a single scale when studying executive functions [12] and are related to the ability to monitor and control compliance with an established plan. A study by Gioia et al. [14] separated these functions and introduced task supervision as a cognitive index scale, with self-monitoring used as a behavioral index scale. For these authors, task supervision is understood as an evaluation of the effectiveness of the supervision, monitoring, and control of one’s work. However, the development of task supervision is incipient in young children, and from the age of seven, becomes a good predictor of achievement in Language Arts and Mathematics. This component is related to maturity and indicates a greater capacity to control situations due to greater experience [42].

The organization of materials allows one to maintain both the area and the materials needed for study in an orderly and organized way. Difficulties in this component often relate to inefficiencies in both private and scholastic life. Learning to organize simple aspects helps improve the achievement of more complex tasks.

Numerous studies have addressed the relationship between executive functions and academic achievement [39,49,56]. Some of these studies are limited to the examination of one or more of the components of this variable [52,57]. On the other hand, as indicated in Gioia et al. [14], the study of executive functions in children has been approached from a cognitive perspective, forgoing behavioral, emotional, and social aspects, in addition to neglecting the evolutionary factors in this population. These authors recommend “leaving the traditional environment of a neuropsychological assessment and obtaining data from the everyday environment of the person being assessed” (p. 7). For this reason, it is important to examine the executive functions during childhood since a large part of the improvements in these functions occurs after the age of 5, with significant implications for scholastic achievement and emotional control [58].

Previous research has indicated that executive functions are related to scholastic achievement starting from the age of 3 years [17] and that, in general, this relationship is significant, influential, and persistent up to the age of 12 years [57,59]; some have even observed a strong correlation at 8–9 years of age [41]. Nevertheless, some authors have concluded that executive functions have a homogeneous composition between 2 and 6 years of age [60], while others found an individualized composition that was separate and distinguished between working memory, inhibition, monitoring, and cognitive flexibility [57]. Some have also found that the relationships between the components of executive functions and academic achievement vary with age, along with the importance of each function in different areas [57,59]. Blair and Razza [32] concluded that the influence of executive functions on educational achievement depends not only on academic competence but also on other factors such as general intelligence.

Therefore, there is scientific agreement that executive functions and their related skills (recall, self-control, planning, and flexibility of thought) are essential to academic achievement [61]. However, despite an increase in the number of studies published on the subject, various limitations were identified in previous studies. Traditional research has been based on aspects such as planning, inhibition, flexibility, and working memory without considering, e.g., emotional or behavioral factors and the developmental processes that all these components present in the child population with different rhythms of maturation [14].

The aim of this study was to analyze the relationship between executive functions and academic achievement in children aged 6 to 9, given the importance of executive functions in studies of the last decade as a predictor of academic success [53,62]. Furthermore, we specifically investigated which components of this factorial structure (scales of the behavioral, emotional, and cognitive regulatory index) have greater predictive weight and if the executive functions (inhibition, self-monitoring, flexibility, emotional control, initiative, working memory, planning, task supervision, and organization of materials) can be linked not only as general achievement cognitive variables but also understood as essential variables for the specific. To this end, we analyzed their relationship to specific areas such as mathematical and linguistic achievement since these subjects are considered relevant to school success by providing the instrumental basis of knowledge. The following hypotheses were proposed: We expected to find a positive and significant relationship between the global level of executive functions and academic achievement. We expected to find a greater relationship of working memory, inhibition, and flexibility with overall academic achievement, and, in addition, we expected working memory to have a particularly strong relationship with Mathematics. However, previous studies have already indicated that there could be variations depending on the specific ages of the subjects in the sample [53]. It is thus important to study a wide age range since the various psychological (attention, memory, and temporal organization) and anatomical processes occur during aging as the frontal lobe develops [63]. All these factors can help us understand the executive functions as a set of multiple and distinct processes. Significant improvements occur during the school years, and inadequate executive function is very likely to explain poor scholastic achievement.

## 2. Materials and Methods

### 2.1. Sample

The sample under study comprised 133 elementary students between the ages of 6 and 9, whose distribution by academic year was as follows: 35.3% in the first year of Elementary Education, 31.6% in the second year of Elementary Education, and 33.1% in the third year of Elementary Education. All of the students attended a subsidized center in the city of Zaragoza (Spain). The socio-economic level of the families was medium-high, with average household salaries of between EUR 36,000 and 42,000 per year as reported by parents. The latest report of the Spanish National Institute of Statistics (2016) put the average income of Spanish households at EUR 26,092 per year. For the distribution of the sample according to sex, 47.4% were girls and 52.6% were boys. The average age was 7.54 years, and the standard deviation was 0.95. The values for academic achievement (they can be evaluated between 0 and 10) were obtained from the average grades of all the subjects taken in an academic year. These values ranged from 5 to 9.4 (M = 7.88; SD = 0.94). Finally, it should be noted that the sampling method was incidental with a convenience sample. The sampling strictly followed the ethics protocol of Committee on Research Ethics in the Autonomous Community of Aragon CEICA (reference no. 04/2019; 27 February 2019). The collaboration of the schools and families was requested in the first instance through a letter of collaboration. Subsequently, the informed consent of the parents was collected alongside consent from the minors. In both documents, the experiment was detailed along with the contact information of the reference researcher.

### 2.2. Measures

Overall academic achievement was evaluated on the basis of the average grades (min. 0; max. 10) obtained by the students in various subjects (Mathematics, Language Arts, Social Sciences, Natural Sciences, Artistic Expression, and English) during the 2018–2019 academic year. The Behavior Rating Inventory of Executive Function, Second Edition (BRIEF 2), in its Spanish adaptation by Maldonado et al., was used to measure executive functions [14]. Raw scores were obtained on nine scales related to executive functions (inhibition, flexibility, emotional control, initiative, working memory, planning, self-monitoring, and task supervision), and raw scores were also obtained for executive functions in general. The clinical scales were combined into three indices: behavioral, emotional, and cognitive regulation, which in turn were combined as a global index of executive function. This is an individual type of questionnaire that must be answered by the parents, teachers, or caregivers of the children being evaluated. In this case, the questionnaires were answered by the parents (family version). BRIEF-2 presents a questionnaire of 63 items (example: “I constantly get up while performing a task”) using a frequency-based Likert scale response (never, sometimes, frequently). A result with high scores, in any scale, indicates the existence of problems in the area where the scale is included (for example, a high score in inhibition will indicate difficulty in controlling one’s impulses and, therefore, problems in the index of behavioral regulation). The questionnaire’s authors indicated high reliability indices through Cronbach’s alpha, which was shown to be above 0.85 for all values. In the present study, the Cronbach’s alpha value was equal to 0.86 for the overall 9-item scale, which indicates high reliability.

### 2.3. Procedure

First, the research design was developed and approved by the school where the study was conducted. Subsequently, with the acceptance of the collaboration of the educational center, the research proposal was presented to the Research Ethics Committee of the Autonomous Community of Aragon: CEICA, obtaining a favorable resolution (no. 04/2019; 27 February 2019). This committee is in charge of evaluating research projects involving people or personal data from BLINDED University. Afterwards, the information regarding the project was sent to the families along with the informed consent for them to authorize the participation of their children. The confidentiality and anonymity of the data were guaranteed through the development of a coding protocol. The center’s orientation team participated in the development of the tests and provided the tutors for each course involved (first grade, second grade, and third grade of Primary Education). The questionnaires, in the family mode, were completed by the parents or legal guardians of the students in their natural contexts. The correction and scoring of the test were performed via computerized procedures (TEAcorrige).

### 2.4. Statistical Analysis

A descriptive statistical analysis was conducted on the demographic variables of the sample. Subsequently, Pearson’s correlations were developed to observe the possible relationships between executive functions and their factors (inhibition, self-monitoring, flexibility, emotional control, initiative, working memory, planning, task supervision, and organization of materials) and academic achievement, both overall and specific (Language Arts and Mathematics). Finally, different regression models were applied to examine the predictive power of the executive functions and different scales or factors in different relationships with general and specific academic achievement. It should be noted that in the models tested, the dependent variable was general academic achievement, with specific achievement in Language Arts and specific achievement in Mathematics. The analyses were carried out using the statistical software IBM SPSS Statistics 25.0 [64].

## 3. Results

First, descriptive statistics were obtained, and correlation analyses were carried out between general academic achievement (GAA); average grade in Mathematics, Language Arts, Social Sciences, Natural Sciences, Artistic Expression, and English; academic achievement in Language Arts (AAL); average grade in Language Arts; academic achievement in Mathematics (AAM); and average grade in Mathematics, along with the scores on general executive function (EF) and each of the factors that compose EF. Neither sex nor age yielded significant results in the correlational analyses with respect to general executive functions (*p* = 0.629 and *p* = 0.498, respectively). The same occurred when performing a correlation analysis between sex and age and each of the executive factors, obtaining significance values greater than 0.05 (Initiative *p* = 0.898, *p* = 0.087; Working memory *p* = 0.942, *p* = 0.549; Task monitoring *p* = 0.197, *p* = 0.168; Organization of material *p* = 0.256, *p* = 0.980; Flexibility *p* = 0.162, *p* = 0.242; Emotional control *p* = 0.526, *p* = 0.240; Inhibition *p* = 0.776, *p* = 0.275; Self-monitoring *p* = 0.544, *p* = 0.811). However, to confirm these data, we proceeded to comparative ANOVA analyses, finding no significance between general executive functions and specific factors in relation to sex and age (*p* > 0.05), and thus these variables were discarded from the analyses. As shown in Table 1, significant correlations were found between general academic achievement and executive functions (r = −0.392, *p* < 0.01), specifically for executive initiative factors (r = −0.272, *p* < 0.01), working memory (r = −0.512, *p* < 0.01), planning (r = −0.402, *p* < 0.01), task supervision (r = −0.531, *p* < 0.01), and organization of materials (r = −0.329, *p* < 0.01). However, when the general executive functions were analyzed with respect to specific achievements such as those in Language Arts and Mathematics, identical significant relationships were found for both disciplines (r = −0.361, *p* < 0.01).

Therefore, the scales that visualize significant relationships for all types of academic achievements studied belong to the index of cognitive regulation according to the model of executive functions linked to the measurement questionnaire. We also observed that the executive functions and their components presented a slightly higher correlation with overall academic achievement than with specific achievements. However, although marginal, the correlation values were slightly higher for academic achievements in Mathematics than for general achievements in both initiative (r = −0.284, *p* < 0.01) and planning (r = −0.406, *p* < 0.01). In addition, following on from non-significant differences, task supervision was more relevant for general academic achievement (r = −0.531, *p* < 0.01) than working memory (r = −0.512, *p* < 0.01), but working memory was more relevant for specific achievements, such as those in Mathematics (r = −0.505, *p* < 0.01) and Language Arts (r = −0.475, *p* < 0.01) than task supervision. However, task supervision offered better results for Language Arts (r = −0.455, *p* < 0.01) than for Mathematics (r = −0.446, *p* < 0.01).

To examine the predictive power of general executive functions on general academic achievement and on specific academic achievements in Language Arts and Mathematics, we performed three linear regressions using the step forward method, taking these variables as the criterion variables for each of the predictive models. Overall, executive functions accounted for 14.7% (GAA), 12.3% (AAL), and 12.2% (AAM) of the variance in student achievement (Table 2). Therefore, higher levels of executive functions positively impacted overall academic achievement, as well as other specific achievements, such as those in Language Arts and Mathematics.

Subsequently, broken down into factors, the predictive power of executive functions on general academic achievement and on specific achievements in Language Arts and Mathematics was analyzed. As shown in Table 3, on this occasion and for the three regression models using the step forward method (where the five scales that correlate with the types of achievement studied (initiative, working memory, planning, task supervision, and organization of materials) were taken into consideration), only the executive factors of task supervision and working memory were significant, as they were able to predict the different global (32.5%) and specific (AAL-25.5%; AAM-27.1%) achievements. In this case, the predictive power for overall achievement was still greater than the ability to explain achievements in specific competencies.

It should be noted that the executive functions as a whole offered less predictive value for academic achievement than the model that only included monitoring of the task and working memory. Therefore, these factors and scales of executive functions remain the most important, regardless of the type of competence, for academic achievements at 6 to 9 years of age.

## 4. Discussion

The purpose of this study was to analyze the relationship and predictive role between executive functions and their components (initiative, working memory, task monitoring, organization of materials, flexibility, emotional control, inhibition, self-control) and academic performance, both globally and specifically in the areas of Language Arts and Mathematics, in 133 students aged 6 to 9 years. Thus, the main findings are that cognitive executive factors are more relevant than behavioral and emotional factors for the prediction of academic achievement (both globally and specifically in Language and Mathematics). Secondly, within the scales of the cognitive regulation index, the best predictors of overall performance, as well as those specific to Language and Mathematics, were task monitoring and working memory. Thirdly, executive functions, in their general or specific measures, are more influential on general academic achievement than on specific ones. This difference, however, is of a marginal nature, and a future study should be conducted to expand on this issue [26]. Therefore, and in relation to the hypotheses of this research, it can be said that the first hypothesis is fulfilled by establishing a positive and significant relationship between the global index of executive functions and academic performance, both general and specific for Language Arts and Mathematics. The second hypothesis of the research is only partially fulfilled since only working memory emerges as a strong executive factor in the prediction of academic performance and not flexibility and inhibition. Finally, the third hypothesis would be fulfilled since it is shown that working memory is capable of significantly predicting both general academic performance and specific academic performance, specifically in the area of mathematics and Language Arts. Finally, the implications of these results in an educational context refer, above all, to the assessment of the level of executive functions at an early age as a basic cognitive factor for proper personal growth. However, the discussion of these results is presented below, and is specified in the conclusions section.

Executive functions have become an important basis for studying children’s behavior and learning, having shown strong validity in predicting academic achievement [65]. Previous research has concluded that cognitive skills are strong predictors of learning behaviors in children aged 7–11 years [61]. The results of the present study also found significant relationships between executive functions and overall academic achievement, as well as in the specific domains of Language Arts and Mathematics [17,37,39,55]. However, some, but not all, of the factors that make up this index are also related to all the types of achievements studied, specifically those that make up the cognitive regulation index (initiative, working memory, planning, task supervision, and organization of materials). In this sense, working memory and planning are developed in infants from age 5 and play an important role in later academic achievement [58]. In addition, planning maintains a greater relationship during the ages of 8-9 years and decreases slightly until adolescence. This factor is linked to achievements in Language Arts according to Sesma et al. [55] and in Mathematics according to Gerst et al. [38], which is consistent with the present research.

However, it should be noted that both planning and initiative have a greater influence on specific subjects such as Mathematics because of their instrumental power. However, although task supervision is related to general academic achievement more strongly than the other factors indicated above, working memory occupies first place for academic achievements in Language Arts and Mathematics. Studies carried out in the United Kingdom indicate that students between the ages of 7 and 14 who score low in working memory tend to perform below the average expected for their age. Therefore, this executive factor stands out for its importance in academic achievement during these years [40,57]. Similarly, the ability to monitor one’s achievements and behaviors appears to be a mechanism related to executive functions during one’s school years. However, in the monitoring of tasks, a connection with language skills is observed, as the level of development of such skills at age 7 contributes to academic results one year later [66]. This is consistent with the results of this study, where homework monitoring was shown to have a greater influence on outcomes in the area of Language Arts than in the area of Mathematics. Similarly, the existence of a relationship between the ability to suppress interference and the monitoring process contributes to the student’s active participation in the control of task achievement [42].

The scales that comprise the index of behavioral regulation (inhibition and self-monitoring) and emotional regulation (flexibility and emotional control) have not been found to be relevant in explaining academic achievement in students aged 6–9. Some authors have already noted that inhibition (index of behavioral regulation) is a good predictor of academic achievement up to age 7 [25] and that flexibility (index of emotional regulation) is a good predictor from age 11 [38]. However, the division of executive functions into hot and cold has linked the latter to the self-control skills needed in emotional situations but not to the level of education [8,42]. Skills with an emotional component improve with age, and a weak relationship with scholastic achievement is observed up to the age of 9–10 years but is almost imperceptible in the first years of compulsory schooling [67,68]. In this sense, this work agrees with previous studies that the ability to adapt one’s emotions to contexts increases with age [24]. The above result indicates that inhibition seems to develop first, and then other components emerge, such as working memory, flexibility, planning, and organization. That is, the changes produced by age indicate the relevant role of the behavioral regulation index scales up to 7 years of age, followed by those of the cognitive regulation index, which overlaps from 10 years of age with the emotional regulation index scales.

The highest predictive weight in this work for an age range of 6–9 years was found for the scales and factors included in the cognitive index of Gioia et al. [14]. Only working memory and task supervision were good predictors of general achievement, as well as achievements in Language Arts and Mathematics. Tsubomi and Watanabe [51] already noted that working memory develops up to age 10, at which point it reaches adult levels, and that better achievement of this variable relates to higher academic scores in Language Arts and Mathematics in children aged 7 to 9. However, working memory is essential for carrying out activities that implement a sequence of actions, and thus the development of working memory requires the introduction of efficient mechanisms that can restrict information from distractors, ensuring that their capacity is not diminished [51]. This ability to analyze and understand a task does not mean that the students are capable of completing the task efficiently. For task completion, it is necessary to add the capacity of supervision, which allows one to review and assess the execution of the task and thus achieve the desired goal. Therefore, the ability to order and prioritize the information received and evaluate the difficulties and the level of knowledge necessary is relevant in this process [14]. These factors all explain the great significance of the relationship between working memory and the planning and monitoring of a task, as well as organization of the relevant materials [14].

While the relationship between executive function and academic achievement is identical in the specific domains of Language Arts and Mathematics, the same is not true when this relationship is studied in terms of different factors. Although the behavior is the same, the other components of the behavioral and emotional indexes (there is no significance) relate slightly better to the area of Mathematics, with the exception of task supervision. However, working memory and homework supervision also explain the higher percentage of academic results in Mathematics than in Language Arts. Unlike the present study, some authors consider visuospatial memory and working memory to be good predictors of the mathematical ability of students between 6 and 12 years old, and inhibition, flexibility, and planning to be good predictors of academic achievement in general [33,37]. Nevertheless, there is a certain consensus with respect to working memory in terms of its involvement in the basic processes needed for arithmetic calculations [38,52], as well as in the acquisition of reading skills [39,55] and academic achievement in general [36]. The fluctuations in the predictive power of the size of this effect highlights the need to clarify whether, depending on the type of academic achievement, the different executive factors assume greater or lesser importance in their relationship to general or specific achievements.

In general terms, the purpose of this paper was partially fulfilled by confirming the relationship between general executive functions and academic performance. However, the study did not find that each of the executive factors is important for the age range studied, 6–9 years. Previous studies have shown that inhibition develops up to the age of 7 [25] and that self-supervision and flexibility are strongly linked to this function [22]. Furthermore, emotional control is related to flexibility and depends on the maturity achieved. [27] From age 7, other factors, such as task supervision, working memory, planning, organization, and initiative, assume a prominent role, especially in the 6–9-year-old age range [45]. However, there are a few papers that deal specifically with the roles of initiative, task supervision, and organization of materials in academic achievement.

It should be noted that the current research has certain limitations that further work should address. The main limitation is that the small size of the sample does not allow generalization of the data, although these data provide an approximation to this topic, as it is in line with previous studies that have already noted the importance of the different factors of executive functions according to the developmental stage of the student.

However, the measurement instrument BRIEF-2 (used in this research) falls within the scales of evaluation for the behavior of executive functions and represents the frequency of achievement of an objective in everyday environments [38]. In addition, these behavioral rating scales were evaluated by a single person (parent or legal guardian) in a single environment, which may have led to less concrete knowledge of the executive functions. Nevertheless, previous studies have highlighted the predictive usefulness of questionnaires completed by parents and teachers [26,38].

Initially, we tried to verify the relationship between executive functions and their factor structures with general and specific academic achievements as a result of the learning process at a specific age. However, it would be advisable to carry out a longitudinal study throughout the years of Primary Education (6–12 years), which would provide information on the role that each of the factors plays in academic results according to age. It should also be considered that executive functions explain only 35% of academic achievement. Thus, it would be interesting to investigate other variables that can help to predict the remaining percentage of variance. This would better complete our work by providing a meta-analysis on this subject to gather information and developing intervention proposals that can improve the teaching–learning process in Primary Education and the subsequent academic results.

Finally, it should be noted that the present study raises possible theoretical modifications such as the clarification of the existing controversy regarding the executive functions’ inclination to predict general rather than specific performance. However, as observed in this study, when analyzing executive functions as a multifactorial element, some factors present their potential with respect to specific performance. For example, in particular, working memory. This suggests the need for a broader, longitudinal study that, in addition to determining this fact, would allow the generalization of the results and their verification at higher ages. On the other hand, there are practical implications, such as the need to evaluate these executive factors at an early age in order to implement specific work plans that allow for the integral development of the student at a cognitive, behavioral, and emotional level.

## 5. Conclusions

Firstly, it can be concluded that cognitive executive factors are more relevant than behavioral and emotional ones for the prediction of academic achievement (both globally and specifically in Language Arts and Mathematics). The low importance of the behavioral and emotional regulation index scales in relation to the academic results of students from 6 to 9 years old is possibly due to the fact that the behavioral components (inhibition and self-monitoring) and the emotional components (flexibility and emotional control) have an impact at earlier ages or during pre-adolescence [30,49]. All these results should be confirmed with future research and literature reviews for each of the educational stages. For the first years of compulsory schooling, the components and scales used by Gioia et al. [14] to form the cognitive index indicate that early scholastic achievement is a consequence of the improvement of executive functions.

Secondly, within the scales of the cognitive regulation index, the best predictors of general achievement, as well as the specific ones in Language Arts and Mathematics, were task supervision and working memory versus initiative, planning, and the organization of materials. This emphasizes that these factors, unlike others, are involved in updating and transforming data to plan and guide behaviors in processes such as language comprehension and mathematical reasoning [52], as well as in effective monitoring that can detect possible errors in the achievement of language tasks (spelling errors) or mathematics (the omission of arithmetical symbols).

Thirdly, executive functions, in their general or specific measures, have a greater influence on general rather than specific academic achievements. This difference, however, is of a marginal nature, and a future study should be undertaken to expand on this issue [26].

Finally, the implications of these results in an educational context relate, above all, to the evaluation of the level of executive functions at an early age as a basic cognitive factor for proper personal growth. We should also consider the importance of some of the factors of academic achievement with specific learning related to linguistic competence and logical–mathematical thinking. Early detection of atypical development of executive functions can lead to the establishment of specific work plans aimed at improving development and preventing future educational problems.

In short, the present study highlights the relevance of executive functions to scholastic achievement. The importance of the scales of the cognitive regulation index compared to the behavioral and emotional indexes was thus verified. In addition, cognitive regulation scales emerged as better predictors of achievement, task supervision, and working memory. Therefore, a high level in these skills relates to a higher level of school success.

However, a number of limitations should also be addressed by, e.g., using a larger sample size and a more sophisticated mediation test. Finally, as a prospective study, it is recommended that these issues should be studied in depth by means of a longitudinal investigation with a large sample.

## Figures and Tables

**Table 1:** Descriptive and correlated statistics of general academic performance, performance in Language Arts, performance in Mathematics, and general executive functions and their components.

	$\bar{x}$	SE	σ2	Statistical Kurtosis	Kurtosis Deviation Error	1	2	3	4	5	6	7	8	9	10	11	12
1 GAA	7898	1034	1070	0.797	0.417	1											
2 AAL	7713	1327	1762	0.589	0.417	0.88 **											
3 AAM	7721	1377	1897	0.420	0.417	0.916 **	0.870 **										
4 EF	89,310	17,842	318,338	0.330	0.446	−0.392 **	−0.361 **	−0.361 **									
5 Inhibition	12,275	3066	9401	−0.114	0.446	−0.154	−0.179	−0.110	0.727 **								
6 Self-monitoring	5879	2167	4698	0.460	0.446	−0.080	−0.083	−0.056	0.601 **	0.556 **							
7 Flexibility	11,560	2905	8440	0.553	0.446	−0.117	−0.109	−0.089	0.528 **	0.191 *	0.226 *						
8 Emotional control	11,603	3322	11,041	1016	0.446	−0.026	−0.051	−0.024	0.603 **	0.621 **	0.491 **	0.413 **					
9 Initiative	7465	2212	4894	0.177	0.446	−0.272 **	−0.230 *	−0.284 **	0.710 **	0.304 **	0.306 **	0.373 **	0.158				
10 Working memory	11,396	3513	12,346	−0.152	0.446	−0.512 **	−0.475 **	−0.505 **	0.802 **	0.430 **	0.332 **	0.315 **	0.247 **	0.685 **			
11 Planning	11,887	3229	10,431	−0.354	0.446	−0.402 **	−0.367 **	−0.406 **	0.830 **	0.526 **	0.376 **	0.274 **	0.224 *	0.703 **	0.781 **		
12 Task monitoring	8474	2732	7469	−0.444	0.446	−0.531 **	−0.455 **	−0.446 **	0.702 **	0.436 **	0.252 **	0.230 *	0.224 *	0.472 **	0.630 **	0.632 **	
13 Organization materials	8756	2393	5730	0.793	0.447	−0.329 **	−0.274 **	−0.300 **	0.712 **	0.421 **	0.346 **	0.301 **	0.288 **	0.482 **	0.546 **	0.611 **	0.540 **

GAA (global academic achievement); AAL (academic achievement in Language and Literature); AAM (academic achievement in Mathematics); EF (executive functions). * *p* < 0.05; ** *p* < 0.01.

**Table 2:** Linear regression analysis to predict overall academic performance, academic performance in Language Arts, and academic performance in Mathematics across executive functions.

		Adjusted R^2^	df	F	*p*	SE	Β	*t*	*p*
Model 1	EF-GAA	0.147	1	20,749	<0.001	0.974	−0.392 ***	−4.55	<0.001
Model 2	EF-AAL	0.123	1	17,105	<0.001	1.246	−0.361 ***	−4.136	<0.001
Model 3	EF-AAM	0.122	1	17,053	<0.001	1.297	−0.361 ***	−4.130	<0.001

GAA (global academic achievement); AAL (academic achievement in Language and Literature); AAM (academic achievement in Mathematics); EF (executive functions). *** *p* < 0.001.

**Table 3:** Linear regression analysis to predict overall academic performance, academic performance in Language Arts, and academic performance in Mathematics across factors or components of executive functions.

		df	F	*p*	Adjusted R^2^	SE	*β*	*t*	*p*
Model 1EF-GAA	Working memory Task monitoring	2	28,419	<0.001	0.325	0.030 0.039	−0.286 ** −0.354 ***	−2.874 −3.556	0.005 0.001
Model 2 EF-AAL	Working memory Task monitoring	2	20,447	<0.001	0.255	0.040 0.051	−0.301 ** −0.271 *	−2.878 −2.588	0.005 0.011
Model 3 EF-AAM	Working memory Task monitoring	2	22,181	<0.001	0.271	0.041 0.053	−0.362 *** −0.223 *	−3.493 −2.153	0.001 0.033

GAA (global academic achievement); AAL (academic achievement in Language and Literature); AAM (academic achievement in Mathematics); EF (executive functions). * *p* < 0.05; ** *p* < 0.01; *** *p* < 0.001.

## Data Availability

No appliable.
